# Bedload transport: beyond intractability

**DOI:** 10.1098/rsos.211932

**Published:** 2022-03-16

**Authors:** Basil Gomez, Philip J. Soar

**Affiliations:** ^1^ Department of Geography and Environment, University of Hawai‘i, Mānoa, Honolulu, HI 96822, USA; ^2^ School of the Environment, Geography and Geosciences, University of Portsmouth, Portsmouth PO1 3HE, UK

**Keywords:** bedload transport efficiency, bedload transport rate, dimensionless specific stream power, quantile locally weighted scatterplot smoothing, sediment availability, transport regimes

## Abstract

Scrutiny of multifarious field and laboratory data amassed over nine decades reveals four distinct bedload transport regimes and demonstrates the search for a universal formula is a fallacious pursuit. In only one regime, in which the supply of transportable material is unconstrained, does the transport rate in some rivers approximate the expected proportional relationship with dimensionless specific stream power (*ω*_∗_). At the other extreme, transport occurs at or near the threshold of particle motion, and the availability of sediment is regulated by the characteristics of the bed surface. In each regime, there is an underlying variation of transport rates at a given discharge, that is neither obscured by long measurement times nor standardized methodologies, and to properly differentiate them, the bedload size must be known. We show a data-driven relationship based on measurements made over several years, across the entire flow range, that requires no *a priori* specification of the association between the transport rate and *ω*_∗_, can reveal nonlinear trends that may otherwise be masked by omni-present temporal and spatial variability. The demise of the search for a universal formula will be accelerated by the development of idiomatic relations that embrace the specificity of rivers in each transport regime.

## 1. Introduction

Why is it so difficult to predict the rate at which a river transports bedload? Gilbert [1, p. 93] recognized that bedload transport rates depend on the energy available, ‘measured by the quantity of water and the vertical distance through which it descends', and the calibre of material in motion. Almost a century and a half later, the search for a formula relating the bedload transport rate to discharge, the energy gradient (as water surface slope) and some measure of sediment size attained its nadir [2]. Nonetheless, although Bagnold [3, p. 454] could not foresee a time when ‘the natural process of sediment transport by flowing water will be understood in precise dynamical terms’, neoteric equations continue to be advanced [4]; but there is no expectation that they, like their predecessors, will either be universally applicable or embraced. This is unfortunate, because bedload transport provides the major process linkage between the hydraulic and material conditions governing river channel morphology; and being able to predict the transport rate with reasonable accuracy would not only help to elucidate the causes and consequences of changes in river morphology, but also to better inform management decisions that affect river functionality [5].

Numerous studies have emphasized that most predictive formulae compute the maximum probable bedload transport rate [6]. However, there is no *a priori* reason to expect that this is the case, especially if not all particle sizes are being transported according to their relative proportions in the bed [7,8]. Moreover, as sediment is entrained, the nature of the flow changes, and grain and form roughness increase flow resistance and decrease the energy available to transport sediment [1,9,10]. These factors ‘reduce the current to the limit of its efficiency’ [1, p. 94].

In two seminal papers, Bagnold [11,12] equated efficiency with the proportion of stream power used for bedload transport and expressed the overall efficiency, *e_b_*, of the transport process as

1.1 $$e_{b}=\frac{i_{b}\tan\;\alpha }{\omega },$$

where (in mass units) *i_b_* is the transport rate of bedload measured as immersed mass per unit width, *ω* is the stream power per unit width (*ρ U Y S*), tan *α* is a friction coefficient for bed material, approximated by the angle of internal dynamic friction of sand (0.63), *ρ* is the mass density of water, *U* is mean velocity, *Y* is mean depth and *S* is water surface slope. The appropriate efficiency is necessarily less than unity because only a portion of the available energy is expended on transporting bedload [1], thus *i_b_* = *ω* (*e_b_*/tan *α*) [12]. In rivers with a plentiful supply of readily transportable material, efficiency declines as particle size increases, and the transport rate varies as *D*^−1/2^ [3,13], where *D* is particle size. Measurements made in such rivers suggest the average upper limit to bedload transport efficiency is of the order of 0.27–0.3 [13,14]. However, in other rivers, efficiency is moderated by both sediment supply and availability [15]. These factors impose a palimpsest of variability on observed transport rates [16,17]; supply being the sediment weathering and mass wasting deliver to a river, as distinct from those size fractions that are actually available for a river to transport [1].

The environmentally controlled rate at which sediment enters a river system exerts a first-order control on transport rates [1,5,12] and gives rise to a spectrum of channel reach morphologies [18]. Their associated transport regimes encompass low-gradient rivers, in which the bedload has the same size distribution as the bed material and mobile particles can be entrained as readily as they are deposited, and steep channels in which the coarsest sediment moves rarely, if at all [1,5]. However, because bedload particles travel relatively short distances during individual flow events [19,20], the time variations in transport rates observed at a reference section are more likely to be the product of transitory, in-channel changes in sediment availability [21]; related, for example, to the passage of bedforms, scour and fill, the formation/breakup of armour or the injection/exhaustion of sediment derived from proximal sources [16]. Moreover, such short-term time variations may be amplified (over longer time periods) by the passage of sediment waves [17,22].

These time-variant and location-sensitive aspects of the transport process are often accommodated by standardized sampling procedures and lengthy collection periods that are explicitly designed to average out any inherent variability [23–25]. Formulae which were supported by or based on time-averaged, stream-wide data suggested it was possible to compute the rate of bedload transport [5,26], and heterogeneous data collected in other rivers and laboratory flumes appeared consistent with a general empirical relationship [3]. Nonetheless, even though sampling and measurements on diverse rivers have generated a plethora of well-documented records of transport rates [27], and sophisticated active and passive technologies now routinely yield continuous, highly resolved, long-period records of transport activity [28], no definitive relationship between the flow and its ability to transport sediment has emerged.

We submit that the reason for the impasse lies not in a lack of understanding of the processes that govern the transport of bedload but in the failure to acknowledge that not all rivers function in the same transport regime. To support our assertion, we draw on records of transport rates spanning 12 orders of magnitude obtained, over the past nine decades, from channels that range in size from small laboratory flumes to large sand- and gravel-bed rivers, to demonstrate the existence of four distinctive bedload transport regimes. The manifestation of these transport regimes suggests that greater emphasis should be placed on data-driven relationships bespoke to the subject river and also implies that the search for a ‘universal’ formula has always been a Sisyphean task. This is because, although every channel is a part of the same universe, each river is a singularity that transports bedload at rates relative to the available power, the first-order control that sediment supply exerts on transport rates and a unique blend of extrinsic and intrinsic controls on sediment availability and, thence, efficiency.

## 2. Material and methods

Efficiency varies inversely with the size of bedload particles, as the overall rate of energy dissipation involved in the transfer of stress from fluid to solids increases [3,13,15], and to accommodate differences in the median size of bedload, *D*_50_, we employ dimensionless specific stream power, *ω*_∗_, defined as

2.1 $$\omega_{*}=\frac{\omega }{\varrho {\left[(G_{s}-1)gD_{50}\right]}^{3/2}},$$

where *ω* is in force units of N m^−1^ s^−1^, *G_s_* is the specific gravity of the bedload (which was assumed to be 2.65, unless otherwise specified) and *g* is the gravitational acceleration [29]. The same reasoning can be used to derive a dimensionless form of the transport rate, i_b__*_, where

2.2 $$i_{b*}=\frac{i_{b}g}{\varrho {\left[(G_{s}-1)gD_{50}\right]}^{3/2}},$$

and *i_b_ g* also has force units of N m^−1^ s^−1^. There are two conspicuous advantages of this interpretation of the transport rate–flow relationship. First, it permits the particle size of the bedload to be actively taken into account, and secondly, because the denominator cancels out, oblique, coplanar efficiency lines, values of which decrease as the transport rate decreases, can be inserted in any double logarithmic plot of *i_b_*_∗_ versus *ω*_∗_. Accepting that *e_b_*, expressed as a percentage 100 (*i_b_*_∗_ tan *α*/*ω*_∗_), is a general measure of the actual work rate, we make no attempt to collapse data which cover a wide range of transport conditions onto a single curve. Instead, we seek to elucidate how variations in sediment supply and availability manipulate the bounds for bedload transport rates set by flow conditions.

The data we rely on to do this are catalogued in the electronic supplementary material. In cases where velocity and depth were not specified, *ω* was calculated as *ρ g* (*Q*/*W*) *S*, where *Q* is water discharge, and if width, *W*, and slope were invariant, station average values were applied. Unless otherwise stated in the original source, the density of water was assumed to be 1000 kg m^−3^ and the density of sediment (*σ*) to be 2650 kg m^−3^. The immersed weight, which is (*σ*–*ρ*)/*σ* times the dry weight of bedload, was used to calculate *i_b_*_∗_, and if the bedload *D*_50_ was not specified, it was approximated by the *D*_50_ of the subsurface bed material. Some records contain zero values for *i_b_*_∗_, which necessarily do not appear on our double logarithmic plots. However, the transport regimes we delineate are neither influenced by outliers nor sensitive to the different bedload sampling and measuring techniques used in either rivers or flumes. We emphasize this point because, in practice, the proportion of the fluvial sediment load described as bedload is determined by the size fractions in motion that a given apparatus or technique is capable of registering [23].

To demonstrate the use of a data-driven approach, we draw on two high-resolution bedload transport records that are also catalogued in the electronic supplementary material. Bedload transport rates at Station 14 in 3.4 m wide Goodwin Creek, MS, USA, were measured at 1 min intervals in three 0.33 m wide traps, during 18 events between November 1988 and February 1990 [30], and in 7.3 m wide Little Turkey Creek, TN, USA, at 5 min intervals in four 0.71 m wide traps, during 11 events between November 2010 and March 2012 [31]. Both records are supported by concurrent measures of discharge and water surface slope. In Goodwin Creek the bedload *D*_50_ was approximated by the *D*_50_ of the subsurface bed material and in Little Turkey Creek the particle size distribution of the bedload was determined for each event.

The non-parametric statistical technique we use is a quantile variation of LOcally WEighted Scatterplot Smoothing (LOWESS; [32]) and the algorithm we implement solves for a specified quantile [28]: quantiles split the range of a probability distribution into continuous intervals between zero and one. To ‘flex’ the quantile trend line through a dataset, linear quantile regression is performed locally within a moving window (the span), which encapsulates a user-defined proportion of the data values across the range of the predictor variable. Inside the window, data are weighted by distance from the target value of the predictor variable using a kernel function; typically for local regression, the tri-cube weight function is adopted [32]. Densely sampled datasets are a prerequisite for producing good models but, although the data and the parameters chosen for the model fit determine the trend that emerges, the smallest span that provides the smoothest fit is typically arrived at visually, by trial and error. We use a percentile block-bootstrapping method to derive the 95% confidence envelope for the 0.5 (median) quantile flexed with a 0.4 (span) moving kernel window. This involves an iterative process of splitting the (log) range of *ω*_∗_ into five bins and independently bootstrapping the points in each bin to generate new bootstrap sub-samples before recombining and running the quantile LOWESS model; here, we use 1000 iterations to plot the confidence bands. ‘Blocking’ ensures the resampling process equitably covers the entire range of *ω*_∗_.

## 3. Bedload transport regimes

### 3.1. Regime I: high availability

**Figure 1.**
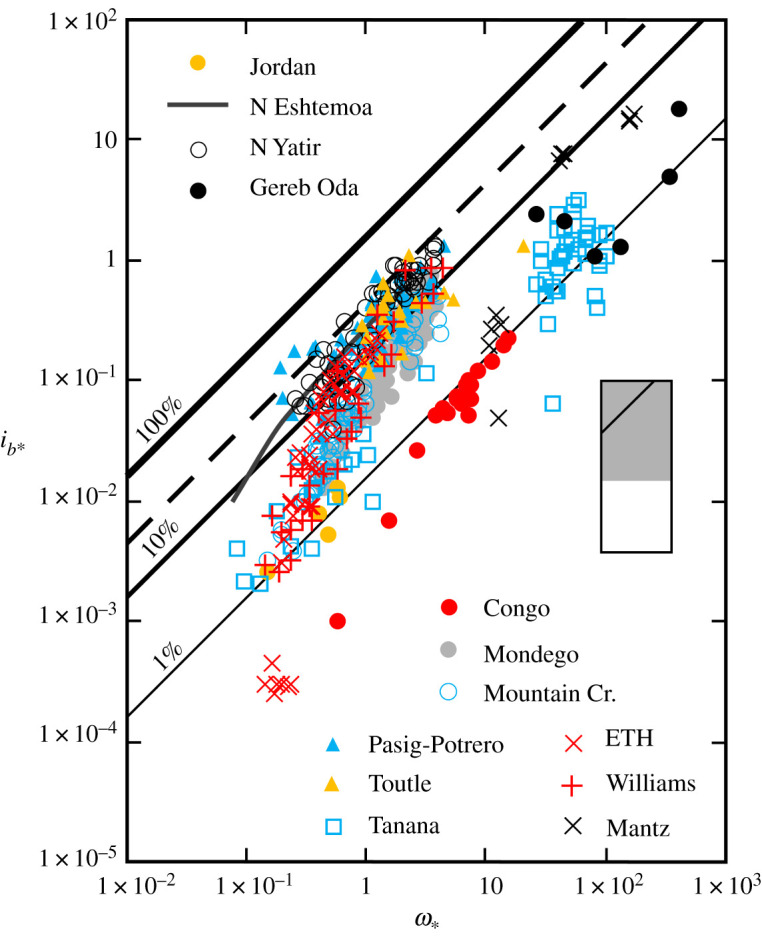
Variation of dimensionless rate of bedload transport (*i_b_*_∗_) with dimensionless specific stream power (*ω*_∗_) in high availability rivers and three laboratory flumes (+ and ×). Least-squares regression (*r*^2^ = 0.93; solid charcoal line) describes the trend in Nahal Eshtemoa, Israel [34]. The family of diagonal lines represent specified bedload transport efficiencies, and the dashed line, the theoretical maximum efficiency (26–30%) attainable in rivers [13,14]. Inset shows relationship of this figure (with 100% efficiency line indicated) to the overall range of the data used to compile figures 1–4, which are catalogued in the electronic supplementary material.

Like others (e.g. [13,14,33]), we recognize that there is a class of rivers which transports bedload very efficiently (*e_b_* approx. 1 to approx. 30%). However, not only does this transport regime embrace a wide range of rivers, but it also encompasses widely referenced measurements made in laboratory flumes (figure 1). In all cases, the transport rate is strongly related to *ω*_∗_ and, in these rivers, high transport rates are sustained by an essentially continuous supply of sediment from upstream or proximal sources, which permits size-selective transport to be maintained even though some size fractions on the bed may either be under-represented or not present in the bedload [7]. Moreover, because the amount of mobile sediment available on the bed is unconstrained by sediment supply, the maximum relative transport rate attained in both rivers and flumes falls within a relatively narrow range (*i_b_*_max_/${\bar{\;i}}_{b}$ approx. 2 to approx. 6), where *i_b_*_max_ is the maximum and ${\bar{\;i}}_{b}$ is the mean bedload transport rate in a given natural or laboratory channel. This range of *i_b_*_max_/${\bar{\;i}}_{b}$ is consistent with the magnitude of temporal variability in bedload transport rates induced by the migration of bedforms [35], which form as individually independent grain steps become less important to the transport process and the particles in motion start to interfere with one another.

If the volume of mobile sediment is less than that required to create equilibrium dimensions, set by water depth, flow velocity and sediment size, bedforms will assume a smaller, supply-limited configuration [36]. However, we have no reason to suppose that a supply limitation is the reason why sand-bed rivers like the Congo River, DR Congo (*D*_50_ = 0.6 mm), Gereb Oda, Ethiopia (*D*_50_ = 0.3 mm) and, in some circumstances (when both components of the sediment load are in the fine to medium sand range), the Tanana River, AK, USA, function less efficiently than, for example, Mountain Creek, SC, USA, (*D*_50_ = 0.9 mm). Gilbert [1, p. 94] noted that ‘every particle which a stream lifts and sustains is a draft upon its energy’, and we construe there are hydraulic controls on the efficiency of rivers with relatively large flow depths, which operate at high transport stages. This is because transport stage, the ratio of the shear velocity to the settling velocity of the sediment, acts as a control on bedform dimensions, such that at high transport stages, bedforms flatten, and the bedload transport rate necessarily declines because a substantial proportion of the mobile material must travel in suspension [12,37]. At a constant stream power, the bedload transport rate also decreases with increasing flow depth, and maximum efficiency requires that the flow depth be a minimum [3,10].

### 3.2. Regime II: limited availability

**Figure 2.**
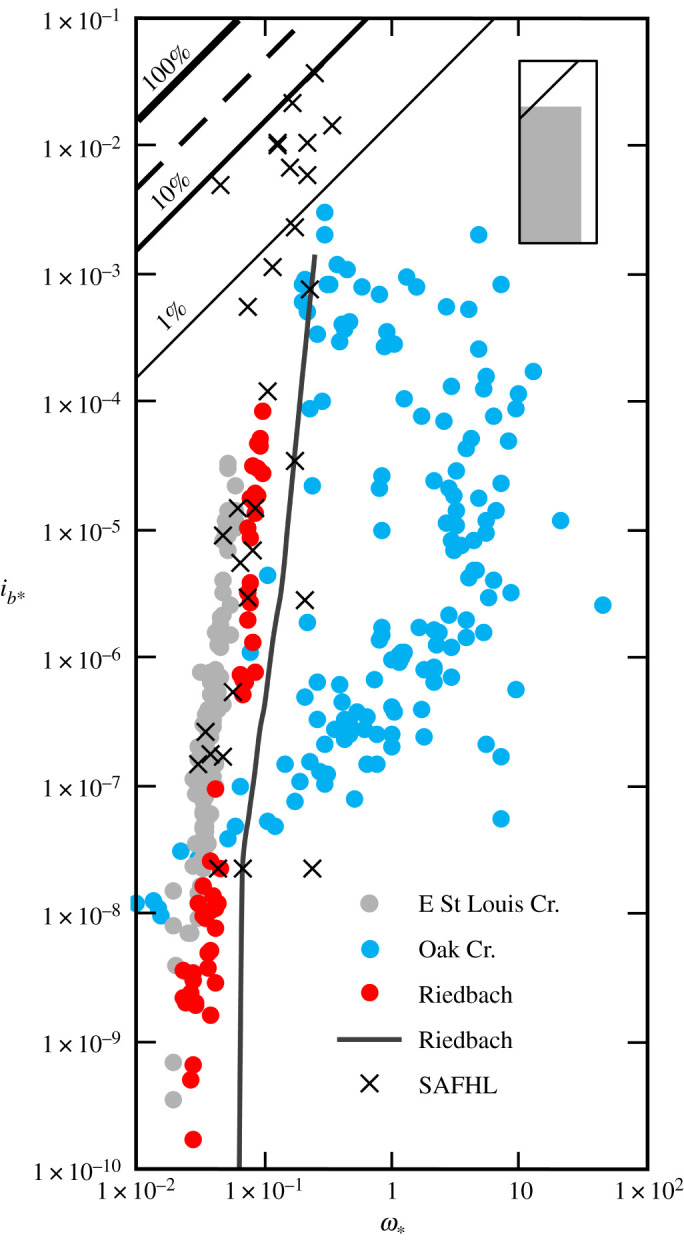
Variation of dimensionless rate of bedload transport (*i_b_*_∗_) with dimensionless specific stream power (*ω*_∗_) in limited availability rivers and a laboratory flume (×). The 0.5 (median) quantile LOcally WEighted Scatterplot Smoothing (LOWESS) curve flexed with a 0.05 moving kernel window (solid charcoal line) describes the trend in the high-resolution data from the steep reach in the Riedbach River, Switzerland (after [28]), and dots are stream-wide mean values for the low-gradient reach [38]. The family of diagonal lines represent specified bedload transport efficiencies, and the dashed line, the theoretical maximum efficiency (26–30%) attainable in rivers [13,14]. Inset shows relationship of this figure (with 100% efficiency line indicated) to the overall range of data used to compile figures 1–4, which are catalogued in the electronic supplementary material.

Rivers and certain flume experiments that operate near the condition at which bedload transport is initiated lie at the other end of the spectrum of transport regimes (figure 2). Comparatively small and highly variable amounts of readily transportable sediment are supplied to these coarse-grained channels, and transport rates are regulated by sediment availability as determined by the response of the typically armoured bed surface to the flow [38,39]. A measurable amount of sediment moves at low values of *ω*_∗_, and as the probability for movement increases, the transport rate increases asymptotically, by as much as six or seven orders of magnitude over the entire *ω*_∗_ range. In Oak Creek, OR, USA, for example, at low *ω*_∗_, sand and fine gravel typically constitute the majority of the bedload, and the transport rate of these size fractions is determined by their availability at the bed surface, which remains intact [39]. As *ω*_∗_ increases, coarser particles begin to be dislodged from the armour, but the bedload transport rate of the finest size fractions declines as sand particles start to become selectively trapped in the bed (‘kinetic sieving’; [40]) or move into suspension. At higher values of *ω*_∗_, the armour breaks up, and there is an increasing tendency towards equal mobility [7].

Headwater rivers like the Riedbach River, Valais, Switzerland, exhibit similar phases of bedload transport; as *ω*_∗_ increases, fine sediment is initially flushed from the stable bed surface, the armour eventually disintegrates and step-pool structures are ultimately effaced [38]. The complex interactions that occur between the driving flow and bed surface limit efficiency and also give rise to zero transport rates across the full range of flows which move bedload in these rivers [28], so that at any given *ω*_∗_, transport rates vary by several orders of magnitude (figure 2). For this reason, we suggest any relationship obtained for such rivers that reduces to a simple power function of the ambient flow conditions is likely to be an artefact of incomplete parameterization [41]. This is because if it cannot be assumed all particle sizes present in the subsurface bed material are in motion, which is a prerequisite in most laboratory experiments [42], then some measure of the size of the bedload is necessarily required to completely characterize the transport regime [6].

Measurements that are directly comparable, because the transport rates were determined using stream-wide or multiple mesh traps and involved long sampling times to eliminate short-term, temporal variability, illustrate the effect (not) specifying the size of the bedload has on our interpretation of the transport rate–flow relationship (figure 2). In Oak Creek, relatively high transport rates at intermediate values of *ω*_∗_ reflect the movement of coarse, unstable particles in the armour [39]. The St Anthony Falls Hydraulic Laboratory (University of Minnesota, USA) flume data exhibit scatter in the relationship between *i_b_*_∗_ and *ω*_∗_ that also arises because the measurements relate to experiments involving five different bed materials [43]. By contrast, the more coherent relationships for East St Louis Creek, CO, USA, and the low-gradient reach of the Riedbach depend on a single, representative bedload size.

### 3.3. Regime III: variable availability

**Figure 3.**
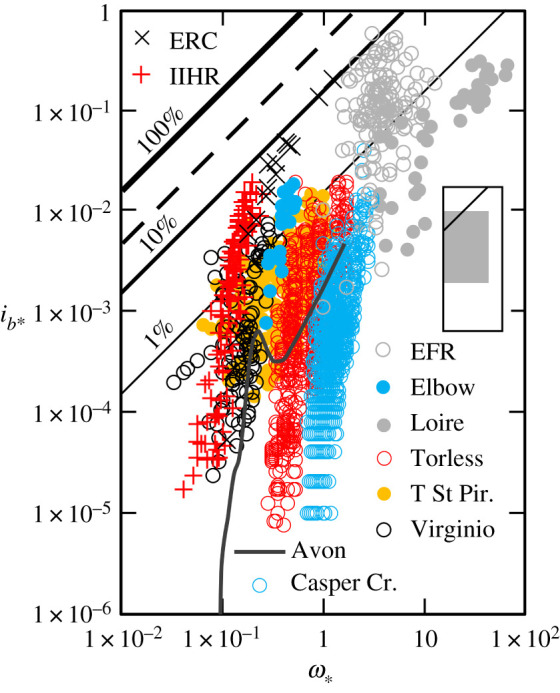
Variation of dimensionless rate of bedload transport (*i_b_*_∗_) with dimensionless specific stream power (*ω*_∗_) in variable availability rivers and two laboratory flumes (+ and ×). The 0.5 (median) quantile LOcally WEighted Scatterplot Smoothing (LOWESS) curve flexed with a 0.05 moving kernel window (solid charcoal line) describes the trend in the high-resolution data from the Avon River, Devon, UK (after [28]). The family of diagonal lines represent specified bedload transport efficiencies, and the dashed line the theoretical maximum efficiency (26–30%) attainable in rivers [13,14]. Inset shows relationship of this figure (with 100% efficiency line indicated) to the overall range of data used to compile figures 1–4, which are catalogued in the electronic supplementary material.

Interposed between the two aforementioned extremes is a heterogeneous class of rivers and laboratory experiments in which transport rates are extremely variable and ordinarily increase rapidly with *ω*_∗_ (figure 3). The pronounced rate variations that define this transport regime accrue from diverse sources, depending on the size of the mobile sediment, degree of bed material stratification and bed morphology. For example, coarse sand and fine gravel in the unarmoured East Fork River, WY, USA, move as bedload sheets, from one storage area into the intervening reach on the waxing limb and on to the next storage area downstream on the waning limb of the snowmelt hydrograph [44,45]. The highest bedload transport rates occur on rising stages, and the relationship between bedload transport and discharge is characterized by clockwise hysteresis. This is also the case in armoured Casper Creek, CA, USA [46]. Although in other rivers, depending on how and when sediment is entrained and transported and the state of the bed surface, different hysteresis patterns have also been observed [47,48]. Measurements from the Loire River, France, and the Environmental Research Centre (University of Tsukuba, Japan) flume exhibit variations in the transport rate that are linked to the movement and architecture of bedforms [49,50]; and in the step-pool Torless Stream, New Zealand, transport rates during and between storms depend on the amount of sediment, derived from gullies and bank collapse, that is held in storage in the channel [51]. Regardless of source, in all cases, sediment availability varies across the entire range of *ω*_∗_.

In circumstances where there is abundant mobile material upstream from a reference section, or additional sediment is recruited as the areal extent of the active bed expands when, for example, the upper surfaces of bars are submerged, these rivers are able to transport sediment relatively efficiently at all but the lowest values of *ω*_∗_ (figure 3); to the extent that, in some channels, the transport rate attains a proportionality with *ω*_∗_. Although, again we caution that in our frame of reference (a lack of) coherence in the transport rate–flow relationship inevitably depends on whether or not the ambient size of the bedload is specified, as in the case of the East Fork and the Loire rivers, or all particle sizes present in the subsurface bed material were, as in the flume experiments, in motion. In other circumstances, irrespective of *ω*_∗_, as reserves are drawn down or depleted, constraints on the availability of readily mobile material reduce the amount of bedload transport that occurs and moderate efficiency.

### 3.4. Regime IV: restricted availability

**Figure 4.**
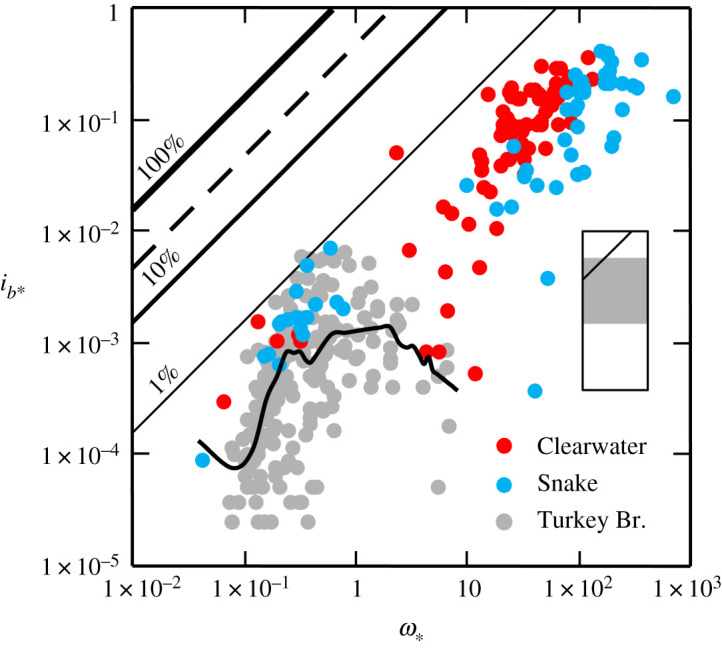
Variation of dimensionless rate of bedload transport (*i_b_*_∗_) with dimensionless specific stream power (*ω*_∗_) in restricted availability rivers. The 0.5 (median) quantile LOWESS (LOcally WEighted Scatterplot Smoothing) curve flexed with a 0.25 moving kernel window (solid black line) accentuates the trend in the high-resolution data from Turkey Brook, Greater London, UK (after [28]). The family of diagonal lines represent specified bedload transport efficiencies, and the dashed line the theoretical maximum efficiency (26–30%) attainable in rivers [13,14]. Inset shows relationship of this figure (with 100% efficiency line indicated) to the overall range of data used to compile figures 1–4, which are catalogued in the electronic supplementary material.

The fourth transport regime we identify pertains to a diverse class of rivers that exhibit a wide range of *ω*_∗_ and a comparatively restricted range of transport rates (figure 4). These rivers can transport bedload very efficiently, but there is often a comparatively poor correlation between the transport rate and flow because, at high *ω*_∗_, the local transporting capability is out of proportion with the availability of potentially mobile material [6]. The effect is clearly manifest in the armoured Turkey Brook, Greater London, UK, where there is a value of *ω*_∗_ at which the transport rate attains its maximum value and, thereafter, apparently no longer depends on local conditions (figure 4; [28]). We infer that this occurs because all the available sediment has been accessed by the flow, and the net result is that the transport rate is unable to increase as *ω*_∗_ increases.

In the armoured Snake River, ID, USA, which, like the Clearwater River, ID, USA, has a high sand throughput load, even at high *ω*_∗_, coarse gravel rarely moves [15], and the stability of these particles limits the movement of the intermediate size fractions, which are typically under-represented in the bedload [7]. By contrast, the finest size fractions appear fully mobile and are nearly always present in the bedload. These size fractions overpass the local streambed gravels and, at all values of *ω*_∗_, the transport rate depends on their availability [6]. Similarly, for constant hydraulic conditions, in some flume experiments, bedload transport rates have been observed to decline as the sediment supply is reduced [52,53].

We also expect that there are other circumstances in which restricted availability might be manifest. For example, in some rivers (such as Gereb Oda, which we designate as a high availability river, figure 1) during high flows, the bulk sediment concentration approaches the threshold for a dilute hyperconcentrated flow [54]; beyond which point a portion of the sand fraction ceases to be transported as bedload [55], and thereafter the transport rate will necessarily be unable to increase as *ω*_∗_ increases. Conversely, bearing in mind that the velocity required to curtail particle motion is significantly less than that required to initiate it [56], in such circumstances higher than expected transport rates could be recorded at low values of *ω*_∗_ as sand is suddenly released from suspension on the waning limb of the flood hydrograph.

### 3.5. Bedload transport efficiency

**Figure 5.**
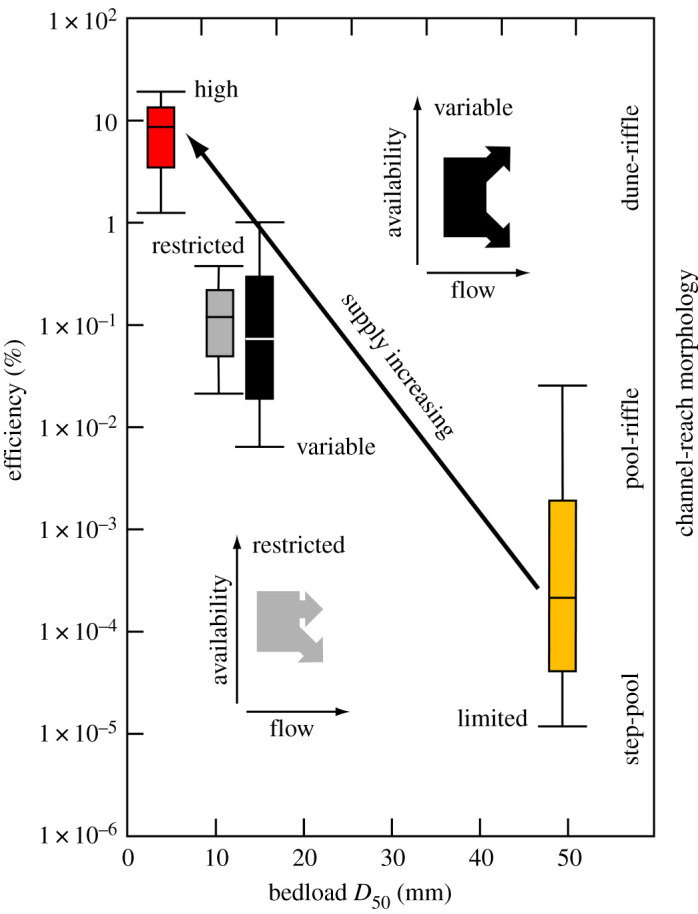
Variation of bedload transport efficiency in relation to aggregate bedload size, channel-reach morphology and the first order control of sediment *supply* in rivers that delineate the four transport regimes (figures 1–4; see text for discussion); the line within each box is the median efficiency, lower and upper boundaries are the 25th and 75th percentiles and lower and upper whiskers are the 10th and 90th percentiles. By contrast, the two insets show alternative trends in sediment *availability* that can occur as the flow rate increases in rivers with a variable or restricted availability transport regime.

To summarize (figure 5), efficiency and the size of sediment in motion are adjusted to the environmentally controlled rate at which sediment is supplied to a river system, which also helps determine channel reach morphology [18]. However, we make a distinction between sediment supply and availability which directly influences the transport rate [21]. High availability rivers are able to transport bedload very efficiently because the bed is fully mobile and there are no constraints on sediment availability [13]. For this reason, there is comparatively little variation in the range of efficiencies observed in high availability rivers. The efficiency of coarse-grained, limited availability rivers is appreciably lower and inherently more variable because transport occurs at or near the threshold for motion, and the availability of mobile material is controlled by bed configuration and stability [26,38]. In variable availability rivers, efficiency is sensitive to transient changes in the availability of mobile material, that are a product of how and when bedload is entrained, the degree of bed material stratification and bed morphology [35,48,57]. The momentum expended on morphological drag owing to immobile boulders, steps and gravel-bed forms, including armour, can also moderate efficiency in both limited and variable availability rivers [1,38,58–60]. Restricted availability rivers have the potential to transport bedload more efficiently, but at high and ofttimes, more moderate values of *ω*_∗_, armouring and exhaustion (and theoretically a change in the rheology of the flow [55]) may limit the availability of mobile material [15].

We now proceed to examine the implications these four transport regimes have for the definition of relationships between hydraulic variables, sedimentological parameters, and the rate at which bedload is transported.

## 4. Wither prediction?

### 4.1. Quantitative mysteries

The bedload transport data we reference encompass at-a-point samples collected in sand-bed rivers that are hundreds or thousands of metres wide, stream-wide measurements made in gravel- or boulder-bed rivers that are only a few metres wide, and experimental data. Collectively, these contrasting datasets provide no evidence for a general empirical relationship between *i_b_*_∗_ and *ω*_∗_, and we note in passing that Bagnold [3, p. 458] acknowledged ‘no general relation between stream power and sediment transport can be expected if the availability of sediment is limited’. Instead, they define a spectrum of transport regimes that are a product of the hierarchical constraints sediment supply and availability exert on the power that is directly available to transport bedload which, in turn, determines how particles are entrained into the flow and are modulated by energy losses [10,12,55].

Bedload transport rates in some rivers and flumes exhibit a direct proportionality with *ω*_∗_, and, in such circumstances, efficiency becomes constant and scales with grain size [3,13]. This is the case in a few high availability rivers (figure 1), but other rivers are sometimes also able to transport bedload very efficiently (figures 3 and 4), if enough sediment is available for transport [1]. Any bedload transport formula derived using such data might be expected to be a reasonably complete correlation, with residual variability linked to the presence of bedforms. The Meyer-Peter and Müller [58] formula is a case in point, wherein the sediment mixture is characterized by a single effective diameter and reducing multipliers account for the portion of the total discharge responsible for moving the bedload and form resistance. The empirical form of Einstein's formula also describes the rate of bedload transport in Meyer-Peter's experiments with uniform sediment undertaken at the Eidgenössische Technische Hochschule, Zürich (English: Swiss Federal Institute of Technology), as well as in Mountain Creek [5]. In devising his ‘worldwide correlation’, Bagnold [61] drew selectively on measurements made in high availability channels, which were supplemented by fortuitous measurements made in some rivers that periodically transport bedload very efficiently. We note, however, that in these rivers and laboratory flumes, the transport rate was found to depend on excess stream power (*ω* − *ω_o_*), where *ω_o_* is the power required to initiate bedload motion, and the relative roughness of the flow; furthermore, dimensionalizing Bagnold's [10] empirical relation suggests that, for coarse sand, *i_b_* = 0.5 $\omega^{1.5}$ [62].

Parker *et al.* [26] judiciously exploited measurements made in Oak Creek, a limited availability river (figure 2), and in the Elbow River, Alberta, Canada, a variable availability river (figure 3), in an attempt to explicitly represent the influence an armour layer has on bedload transport rates. The effect has also been characterized using two-phase regression relationships [38,63] and models that differentiate between the transport rates of sand and different classes of gravel [64,65]. However, approaches that are sensitive to bed state or grain size distribution are not designed for use in rivers with a predominately sand bedload, and consequently, these and many other formulae characteristically overpredict transport rates [6]. This is because sediment supply and availability constantly manipulate the bounds set by the flow conditions, so there is no semblance of proportionality between the bedload transport rate and the mean, channel-wide hydraulic conditions in many rivers. Thus, despite their universal uptake, and even when conscientiously employed, all predictive formulae unsurprisingly have rather confined applicability; a paradox that continues to prevail largely unchallenged.

We have suggested elsewhere that spatial and temporal variability generated by the various factors that affect bedload transport should not be viewed as ‘noise’ and also emphasized that zero values of the bedload transport rate are real occurrences [28]. This introduces a new dilemma, because high-resolution bedload transport rate records are inevitably noisy and exhibit zero values across a wide, or even the full, range of *ω*_∗_. A related issue concerns the lengthy sampling times that frequently are required to register low transport rates in limited availability rivers [66]; which, in the case of Oak Creek, sometimes extended to four days [39]. In most rivers, the available power is not constant, and defining the hydraulic condition that moves bedload over lengthy periods of time introduces a new source of uncertainty, because a temporal and channel-wide integration is now required to obtain the mean value. When applying a formula, the premise is that a specific relationship exists between this mean value, the (typically) median size of the sediment in motion (which may or may not be known and is often assumed to be constant) and the time-varying rate at which bedload is transported. The task of prediction is further confounded if both width and slope are held constant when deriving the mean value; whence, for all intents and purposes, stream power amounts to discharge in disguise.

### 4.2. Towards a new paradigm

It is clearly not reasonable to expect any formula to perform successfully under arbitrarily varying conditions or in circumstances where the input data are ill-defined and when unanticipated conditions apply [6]. We believe that, rather than continuing to painstakingly distil the essence of bedload transport and mindful of the effect inadequate parameterization has on predicted rates [41], one way to resolve this methodological impasse is to use data-driven approaches to reveal trends, patterns and structure in records which display many possible bedload transport rates for each value of *ω*_∗_. The procedure we employ is analogous to that used to characterize nonlinear relationships between suspended sediment concentration and discharge [67–69]. To illustrate its application, we draw on the high-resolution records obtained from two small rivers that transport variable amounts of sand and gravel and also, for unknown reasons, exhibit different transport rates for rising and falling flow stages [30,31]. Limited-width traps censor some components of the bedload and afford a time-averaged perspective of the particle sizes in motion, and, like other high-resolution bedload transport records, both datasets contain a large number of zero transport rate values and are generally incompletely parameterized with respect to the factors that potentially influence the transport rate. Thus, it is challenging to relate the measured rates to an appropriate flow quantity using conventional, least-squares regression.

As a suitable alternative, quantile LOWESS is a procedure for fitting smooth curves to diffuse arrays of data [70], that combines the core strengths of quantile regression (the ability to account for variability; [71]) and LOWESS (the capacity to accommodate nonlinear trends [32]). The defining feature of quantile LOWESS is that, because no *a priori* assumption is made about the mathematical form of the smoothed curve, ‘it allows the data to speak for themselves’ [70, p. 578], and we have shown previously that it is adept at revealing recondite trends in high-resolution bedload transport records ([28]; figures 2–4). More generally, we note that, inasmuch as the modelled function is derived from input-output pairs, LOWESS is one of a number of widely applied, supervised machine learning tasks that can ‘learn’ information directly from noisy data values without (by relaxing the linearity assumption) relying on a model that is described by a predetermined equation [72].

**Figure 6.**
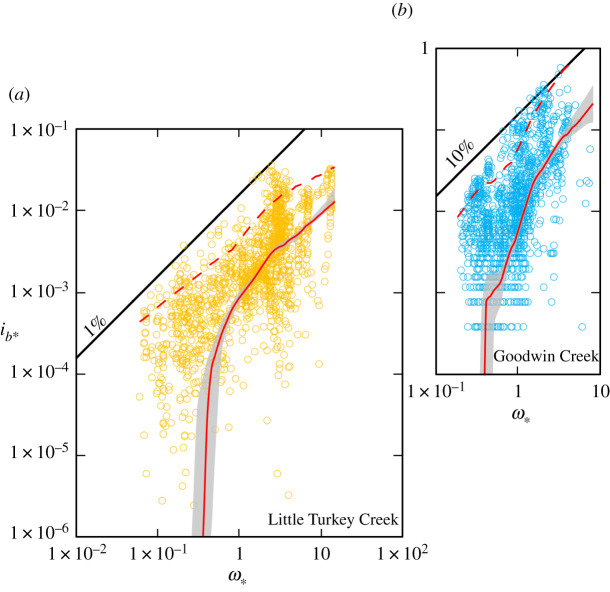
(*a*) The 0.5 (median) quantile LOcally WEighted Scatterplot Smoothing (LOWESS) curve flexed with a 0.4 moving kernel window fitted to Little Turkey Creek, TN, USA, and (*b*) Goodwin Creek, MS, USA, bedload transport data (solid red lines). Grey shading delineates the 95% confidence envelopes computed by percentile block-bootstrapping. Dashed red lines are 0.95 quantile LOWESS curves flexed with a 0.7 moving kernal window. Diagonal lines are specified bedload transport efficiencies. See text for discussion.

We refer to a commonplace measure of central tendency, but there is no intrinsic reason to exclusively use the 0.5 (median) quantile, because a relationship can be computed for any quantile. The 0.95 quantile, for example, might be used to delimit the envelope that defines the upper limit to transport in a particular river. However, because gaps that are the result of uneven data acquisition often emerge near the edges of a data cloud, for high and low quantile estimates, a larger span will often be required to remove most of the roughness in the curve. It is for this reason that the 0.95 quantile LOWESS trend portrayed in figure 6 is flexed with a 0.7 moving kernel window.

For computational purposes, zero values in the data were replaced with an infinitesimally small, proxy value (1 × 10^-10^); even so, the bottom portion of the trend lines still descends to zero. Our preference is to retain zero values because, although measurement and sampling times are often lengthened to negate the possibility of recording a zero value, the probability of sediment transport is never zero except in still water [43], and zero values are a prominent component of high-resolution datasets [28]. If their occurrence is not random and the number of incidences is high, removing zero values also has the potential to introduce selection bias. That said, we acknowledge that retaining zero values in high-resolution data could be construed as problematic, inasmuch as the bottom part of the curve deviates from the ‘apparent’ trend in the lower, non-zero measurements of bedload transport rates that are visible on our double logarithmic plots (figure 6). However, some empirical bedload transport models also predict that, in gravel-bed rivers with an appreciable sand load, the transport rate becomes vanishingly small at low values of *ω*_∗_ [26,73], and their application to Little Turkey Creek shows the same trend with respect to the lower range of measured non-zero transport rates as illustrated by our 0.5 (median) quantile LOWESS curve [31]. The implication is that, in cases where zero values feature prominently in the measurement record, one's perception of a bedload transport flow–relationship especially, but not exclusively, at low values of *ω*_∗_ may be somewhat tarnished by an inability to follow the data where they go and visualize values that are mathematically impossible to plot on a logarithmic scale.

Quantile LOWESS can be used to depict the local relationship between a response and predictor variable (in this case *i_b_*_∗_ and *ω*_∗_) over portions of their ranges, but we are more interested in the form of the entire curve. This is because bedload transport is a continuous process that exhibits considerable temporal variability [74]; and, like Einstein [5] and Bagnold [10], we believe it should be possible to construct an overarching relationship that reflects the environmentally controlled rate at which sediment is supplied to a river. To the extent that if the movement of bedload can be demonstrated to be a function of discharge (or *ipso facto* some other driving force), then the sequence of discharges is unimportant, and it should be possible to determine the annual transportation through a reference section from the duration curve of the flow [5]. The high-resolution bedload transport records from Goodwin and Little Turkey creeks, which not only delimit the range of transport and flow conditions experienced in these rivers but also cover a reasonable span of elapsed time, are illustrative of the type of data required to accomplish this task.

The application of quantile LOWESS permits a functional dependence to be evaluated in cases where the data points do not form a readily discernible pattern, as is often the case with high-resolution bedload transport records [28]. The fitted relationship provides a useful alternative to the ‘linear’ forms (e.g. a power-law trend fitted by linear regression to log-transformed variables) that conventionally have been used as a basis for estimating the bedload yield within a range of observed discharges [38,63,75]. However, we emphasize that it is not a universal substitute for conventional statistical methods, which may be extrapolated as well as interpolated and have a demonstrable use in circumstances where the adoption of standardized sampling procedures averages out some temporal and spatial variability and a power-law trend is especially pronounced [76]. Having the ability to choose between different procedures is potentially important because, even in high availability rivers, there is ofttimes curvature (in logarithmic space) of the transport rate – flow relationship ([10]; figure 1), and integrating bedload over a cross-section or computing bedload from cross-section-averaged data has the potential to generate singular results [6,77]. It is also well established that different shapes of suspended sediment concentration–water discharge relationships give rise to differences in the long-term average yield and event yield magnitude–frequency relationships [67], and we have no reason to suspect that this is not also the case for bedload.

An unresolved question is to what extent should a predicted bedload transport rate conform with a measured rate [41]? We are unaware of any mechanism for estimating the bedload transport rate for a given flow condition, derived from field or laboratory measurements, that does not relate, albeit often unintentionally, to one or, by design, more (e.g. [4]) of the transport regimes we have identified. However, formulae are habitually applied without regard to the circumstances for which they were derived [6]. We suggest that in high availability rivers, where there is only a narrow range of inherent variability in the transport rate at a given *ω*_∗_ (figure 1), it should be possible to compute temporally representative bedload transport rates for a range of flows to within one order of magnitude. The theory governing bedload transport in these rivers is well established [5,12,58], but a methodology for determining the exact proportionality between the transport rate and stream power or any other driving force remains to be elaborated. In limited availability rivers, the flow responsible for the initiation of grain motion and the state of the bed surface also needs to be taken into account. Methodologies exist for doing this [26,73], but their veneer of respectability fades once it is appreciated that, because lower flows do not invariably transport smaller bedload sizes and *vice versa*, a complete relationship necessarily requires the size of the bedload to be specified (figure 2). In particular, it remains to be seen how variations in the critical condition for motion in a given river owing, for example, to the presence of an armour layer, hiding etc., can be accommodated, so that bedload transport rates can be reliably computed over the entire range of flows that are capable of mobilizing and sustaining bedload transport in these rivers. The large uncertainties these factors engender suggests to us that it might not be possible to compute the mean bedload transport rate in limited availability rivers to within one order of magnitude. For variable and restricted availability rivers, the problem is even more complex; inasmuch as the cause of rate variations is linked to the availability of transportable material (figure 5), that can sometimes be difficult to elucidate [30,31,78], or is contingent upon processes remote from a reference section that determine the availability of the sand and gravel in transport [57]. A data-driven approach dispenses with the need to specify the mathematical form of the transport rate–flow relationship and, if high-resolution bedload transport records are available, we believe the 0.5 (median) quantile is a viable basis for computing average bedload transport rates over the wide range of flow conditions that occur in these rivers. The accuracy with which this can be accomplished not only depends on the fidelity of the recorded bedload transport rates but also rests on whether or not all the associated hydraulic parameters which vary with stage (including slope, and width in some large rivers), as well as the size of the bedload, are specified. This is also the case if trends fitted by linear regression to log-transformed variables are to be productively used.

That said, we do not mean to imply that the only requirements needed to move forward are to make as many measurements as possible and apply machine learning techniques to a profusion of data; or, to the extent that LOWESS and other non-parametric techniques do not yield a global solution that can be used to predict values of new data, the fitted functions obtained in such a manner are a substitute for well-grounded theoretical models. At the macroscopic scale, stream power is a convenient proportional (and necessarily averaged) variable that has a definable meaning and can be directly related to the bedload transport rate [6,10–12]; but, at the microscopic scale, bedload transport is a noise-driven process that belies parsimonious description [79]. The key to better explanation may be a greater understanding of the dynamics of the interactions among the system components that generate the complex, irregular behaviour [80], and particle-based approaches (see [81]), which decouple bedload statistics from the fluid (water) flow, may eventually yield robust estimates of transport rates. Accordingly, we suggest a data-driven approach, which can be applied to long time series of near-continuous measurements of sediment flux, may help isolate patterns that must necessarily be explained in physical terms [82].

Assessing the response fluvial systems have to climate or anthropogenic change, the science and practice of river restoration, and the development of sustainable approaches to river management all require knowledge of transport rates over the range of flows that are capable of mobilizing and sustaining the motion of bedload. However, the ability to reliably estimate the amount of bedload transported by a given flow, or compute bedload yield, is often an unfulfilled component of geomorphological, engineering and ecological investigations of rivers. The overarching thesis of this paper is that no new transformative knowledge is required to rectify this state of affairs in the immediate future, because the necessary paradigm shift is simply contingent upon the more focused application of extant knowledge and practices.

## 5. Conclusion

A stream power-based approach is an appropriate perspective from which to examine the association between measured transport rates and the transporting flow [10]; but there are innumerable rivers in the universe of fluvial systems and, contrary to prior optimistic thinking [61], we see no reason to assume that a generalized bedload formula or universal empirical correlation will ever emerge. The four transport regimes we have identified, by drawing on multifarious field and laboratory records of bedload transport rates amassed over nine decades, are consistent with the knowledge that alluvial channel morphologies reflect the sediment supply and the power of the river at the point of supply [1,18]. However, the ambient transport rate is determined by sediment availability [21].

In high availability rivers, the bed is fully mobile, and there are no constraints on sediment availability because there is a continuous supply of readily transportable material. These rivers transport bedload very efficiently across the entire range of *ω*_∗_ (figure 1). The efficiency of limited availability rivers is appreciably lower (figure 2), because bed configuration and stability regulate the availability of those fractions of the bed material on which the transport rate depends. In variable availability rivers efficiency is a product of how and when bedload is entrained, the degree of bed material stratification and bed morphology (figure 3). To the extent that, in some circumstances, these rivers function very efficiently, whereas at other times, constraints on sediment availability reduce the amount of bedload transport that occurs and moderate efficiency. Restricted availability rivers have the potential to transport bedload very efficiently (figure 4), but even at moderate values of *ω*_∗_, the availability of material in transport as bedload is known to be constrained by the state of the bed surface and/or exhaustion and may, in theory, be influenced by the rheology of the flow.

In general, efficiency is commensurate with the size of the bedload, as prescribed by the environmentally controlled rate at which sediment is supplied to a river system and becomes available for transport ([1]; figure 5). It has been suggested that ephemeral rivers are more efficient at transporting sediment than their perennial counterparts [14,83]. However, inasmuch as the availability of transportable material is the principal determinant of efficiency, in our dimensionless frame of reference, any such differentiation appears unwarranted (figure 1).

The temporal distribution of bedload transport is a complex, nonlinear function of sediment availability, fluid forcing and bed state, and the data required to cement the connection between the measured transport rates and flow conditions are often lacking [41]. Rather than continuing to meander between randomness and determinism, we suggest that a data-driven approach can provide an informative graphical and mathematical description of the transport rate–flow relationship (figure 6). Although it may have particular use when applied to variable and restricted availability rivers, where the mechanisms that give rise to rate variations may be difficult to identify, a data-driven approach is potentially applicable to any transport regime. Always assuming that the measurements, regardless of how they are obtained, delimit the range of transport and flow conditions experienced in a river and cover a reasonable span of elapsed time, then the picture that emerges of the underlying trend and its associated variability can be used to inform a bespoke bedload transport relationship and reinforce theory. A wide range of geomorphological, engineering and ecological issues could be addressed by applying the information contained in such a relationship. Thus, we conclude that efforts to forecast bedload transport rates have now reached a tipping point. Researchers will either continue to believe that ‘process based’ and/or ‘theoretical’ explanations will eventually provide a ‘universal’ solution to the conundrum of predicting transport rates (for a given stream power or another control variable) that is applicable to a profusion of conditions. Or, we can collectively anticipate that the elaboration of data-driven relationships (derived using either conventional or modern statistical techniques) will help motivate research into representative models for bedload transport that are able to embrace the specificity of rivers with respect to the transport regimes we have identified, thereby bringing an end to the search for a universal equation; a panacea that (we contend) does not exist.

## Data Availability

The information relied upon is catalogued in the electronic supplementary material.
